# Bacterial Cellulose–Silk Hydrogel Biosynthesized by Using Coconut Skim Milk as Culture Medium for Biomedical Applications

**DOI:** 10.3390/gels10110714

**Published:** 2024-11-06

**Authors:** Junchanok Chaikhunsaeng, Phasuwit P. Phatchayawat, Suchata Kirdponpattara, Muenduen Phisalaphong

**Affiliations:** 1 Bio-Circular-Green-Economy Technology & Engineering Center, BCGeTEC, Department of Chemical Engineering, Faculty of Engineering, Chulalongkorn University, Bangkok 10330, Thailand; junchanok.ch@gmail.com; 2 Biomedical Engineering Program, Faculty of Engineering, Chulalongkorn University, Bangkok 10330, Thailand; phasuwit.p@gmail.com; 3 Department of Chemical Engineering, Faculty Engineering, King Mongkut’s University of Technology North Bangkok, Bangkok 10800, Thailand; suchata.k@eng.kmutnb.ac.th

**Keywords:** bacterial cellulose, silk, coconut skim milk, biopolymer, tissue engineering, wound dressing

## Abstract

In this study, hydrogel films of biocomposite comprising bacterial cellulose (BC) and silk (S) were successfully fabricated through a simple, facile, and cost-effective method via biosynthesis by *Acetobacter xylinum* in a culture medium of coconut skim milk/mature coconut water supplemented with the powders of thin-shell silk cocoon (SC). Coconut skim milk/mature coconut water and SC are the main byproducts of coconut oil and silk textile industries, respectively. The S/BC films contain protein, carbohydrate, fat, and minerals and possess a number of properties beneficial to wound healing and tissue engineering, including nontoxicity, biocompatibility, appropriate mechanical properties, flexibility, and high water absorption capacity. It was demonstrated that silk could fill into a porous structure and cover fibers of the BC matrix with very good integration. In addition, components (fat, protein, etc.) in coconut skim milk could be well incorporated into the hydrogel, resulting in a more elastic structure and higher tensile strength of films. The tensile strength and the elongation at break of BC film from coconut skim milk (BCM) were 212.4 MPa and 2.54%, respectively, which were significantly higher than BC film from mature coconut water (BCW). A more elastic structure and relatively higher tensile strength of S/BCM compared with S/BCW were observed. The films of S/BCM and S/BCW showed very high water uptake ability in the range of 400–500%. The presence of silk in the films also significantly enhanced the adhesion, proliferation, and cell-to-cell interaction of Vero and HaCat cells. According to multiple improved properties, S/BC hydrogel films are high-potential candidates for application as biomaterials for wound dressing and tissue engineering.

## 1. Introduction

Thin-shell silk cocoon (SC) and bacterial cellulose (BC) are biopolymers synthesized by silkworms and bacteria, respectively. These two biopolymers could be combined as biocomposites owing to their special properties. For example, the BC-silk fibroin sponge scaffold exhibited nongenotoxicity with a high potential for tissue generation [1]. Electrospun silk–elastin-like protein membrane covered with BC promoted skin cell proliferation, a promising biomaterial for skin substitution [2]. BC was functionalized with silk sericin and hyaluronic acid for application as a wound dressing and cosmetic materials [3]. Several modifications have been made for the improvement of important properties of materials applied in biomedical fields, wound dressing, and tissue engineering. The crucial properties include nontoxicity, biocompatibility, biodegradability, absorption ability, appropriate mechanical properties, and antimicrobial activity [4,5,6,7,8].

Silk is popularly known in the textile industry. It is also widely used in medical applications. Silk is obtained from silk cocoons, naturally produced by Bombyx mori silkworms. The leftover byproduct after the process of removing the silk thread fibers from the cocoon is a thin-shell silk cocoon (SC), which is composed of two proteins: fibroin and sericin [9]. Extracts from SC, especially fibroin, have been applied in medical areas such as wound healing, 3D scaffolds for ligaments, and vehicles for drug delivery [10]. Fibroin, a semi-crystalline protein, provides stiffness and strength to the structure of a silk cocoon. Fibroin has been actively studied for applications in the forms of bio gel, film, powder, sponge, fiber, and membrane [11]. On the other hand, sericin is an amorphous protein acting as an adhesive binder to maintain the structural integrity of the fiber and the cocoon [12]. Fibroin is rich in amino acids, glycine, and alanine, whereas sericin contains no fewer than 18 amino acids, including essential amino acids, and is especially rich in serine [13]. Both fibroin and sericin are biodegradable and biocompatible. Due to its high promotion of cell adhesion and growth, fibroin has been extracted from the cocoon and supplemented into other polymers [11,14] using many techniques, i.e., degumming, extraction by solvent dialysis, and centrifugation [1,15], leading to high production cost and waste generation.

BC is a cellulose nanofiber network produced by bacteria such as *Acetobacter*, *Agrobacterium*, *Pseudomonas*, *Rhizobium*, and *Zoogloea* [16]. *Acetobacter* is widely studied and exhibits an incredibly high BC yield, making it suitable for commercial production [17]. BC, nanocellulose, consists of repeating units of glucose molecules [18] linked by beta-1,4 glycosidic linkage. BC has unique properties, including high water-holding capacity, high crystallinity, high hydrophilicity, high tensile strength, highly pure and ultrafine cellulose fiber network structure (free of lignin, pectin, and hemicellulose) [19], nontoxicity, and biocompatibility. In medical fields, BC has been applied as an artificial blood vessel for microsurgery [20], a scaffold for tissue engineering [21], artificial skin for wound healing [22], and a scaffold for cartilage and bone regeneration [23,24]. Natural proteins such as collagen, silk peptide, and silk fibroin have been studied to integrate into BC and polysaccharide composites in order to improve the biocompatibility of biocomposites [25,26,27].

Mature coconut water, a byproduct from coconut milk and coconut oil factories, contains about 5% of the total solids of nutrients, such as various sugars (sucrose, glucose, fructose, and sorbitol), amino acids, vitamins, and minerals [28]. Coconut water is typically used as a raw material for BC production because it promotes bacteria growth and enhances BC synthesis [29]. Another main byproduct from coconut milk and coconut oil factories is coconut skim milk [30]. Coconut skim milk contains about 1% fat and other nutrients, such as protein, carbohydrates, and minerals [31]. Coconut skim milk and mature coconut water have been reported for potential use as a fermentation medium for BC production [32]. Cellulose yield, structure, and characteristics are influenced by the strain of microorganisms, fermentation conditions, and culture medium.

In this study, S/BC hydrogel films were fabricated by a biosynthesis technique using coconut skim milk as culture media in comparison with mature coconut water culture media. The powders of SC were directly supplemented into the medium. The effects of different feedstock and silk concentrations on the mechanical and biological properties of the composite films were examined. The biocompatibility of the composite hydrogel films was evaluated with Vero and HaCat cells. This study aims to develop S/BC hydrogels biosynthesized through a simple, facile, cost-effective method by using byproducts of coconut oil and silk industries as a culture medium for further applications in biomedical areas.

## 2. Results and Discussion

### 2.1. Morphology of Scaffold

**Figure 1:**
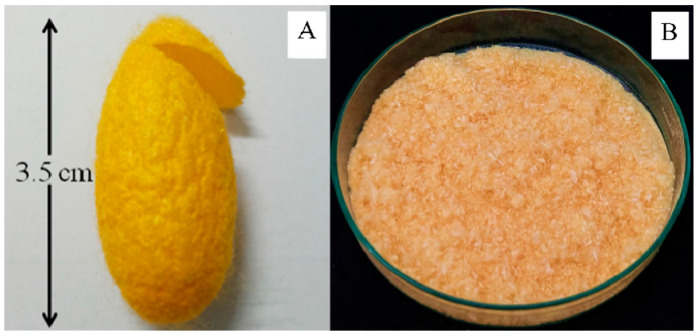
Thin-shell silk cocoon (**A**) and silk cocoon powder (**B**).

**Figure 2:**
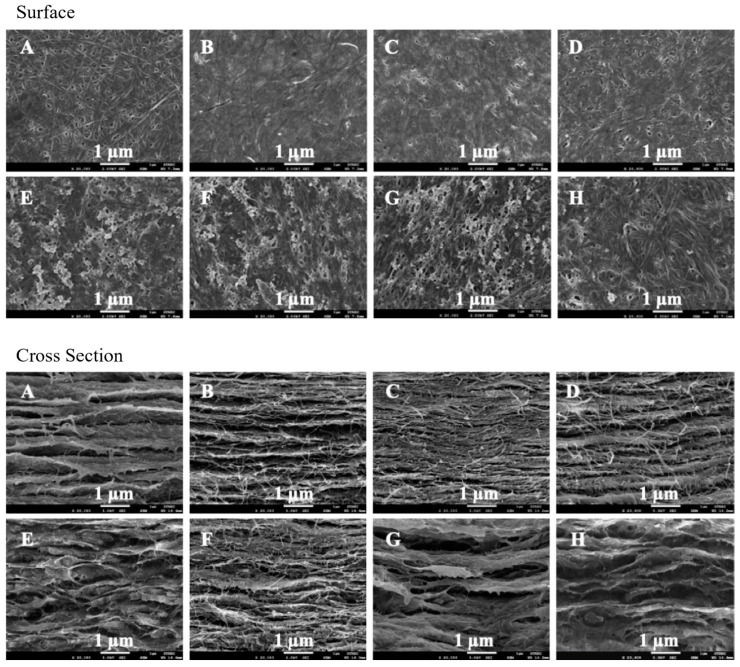
FESEM images of surface morphologies (**top**) and cross-section (**bottom**) at magnification of 20,000× (**A**) BCW, (**B**) 0.10S/BCW, (**C**) 0.15S/BCW, (**D**) 0.20S/BCW, (**E**) BCM, (**F**) 0.10S/BCM, (**G**) 0.15S/BCM, and (**H**) 0.20S/BCM.

A thin-shell silk cocoon (SC) and silk cocoon powder are shown in Figure 1. Surface and cross-sectional images of BC films from mature coconut water (BCW) and BC films from coconut skim milk (BCM) with and without adding silk powders are shown in Figure 2. Without silk supplementation, BCW and BCM were obviously different. BCW surface (Figure 2A, top) presented an ultra-fibrous network structure with a pore size of 0.08–0.1 µm (measured by Image J 1.54k software). For BCM (Figure 2E, top), small white particles all over the surface were observed. Constituents of coconut skim milk were approximately 73.3 g carbohydrate/kg, 42.6 g protein/kg, 8.7 g fat/kg, and 8.5 g ash/kg [31]. The small white particles of coconut skim milk attached to the nanocellulose matrix of BCM film during the BC biosynthesis process. For a cross-sectional view of BCW (Figure 2A, bottom) and BCM (Figure 2E, bottom), sheet layers of cellulose were formed, but more disorders were noticed in BCM. Therefore, particles in coconut skim milk might play an important role in obstructing the arrangement of the cellulose fibers extruded from bacterial cell walls.

Different silk contents (0.1, 0.15, and 0.2% *w*/*v*) were supplemented into BC medium. From Figure 2B–D, good incorporation between BC and silk was observed in S/BCW and S/BCM. Silk particles filled the pores of the BC fiber network and dispersed on the BC surface. The increase in silk loading resulted in a higher amount of silk covering the surface area, as seen in Figure 2C,D, top. This finding agreed well with BC-silk sericin prepared by the in situ method, in which sericin covered the surface and assembled fiber to form a bigger ribbon in the matrix [3]. For S/BCM (Figure 2F–H, top), white particles on the surface were less observed because of the incorporation of silk, particularly on BCM films loading with silk at 0.2% *w*/*v* (Figure 2H, top). For the cross-sectional area, thin layers of BC fibrous nanocellulose sheet incorporated with silk were formed in both S/BCW (Figure 2B–D, bottom) and S/BCM (Figure 2F–H, bottom). Previously, it was reported that a very well-interconnected porous structure was formed in the BC/silk fibroin sponge scaffold [33].

### 2.2. Mechanical Properties

**Figure 3:**
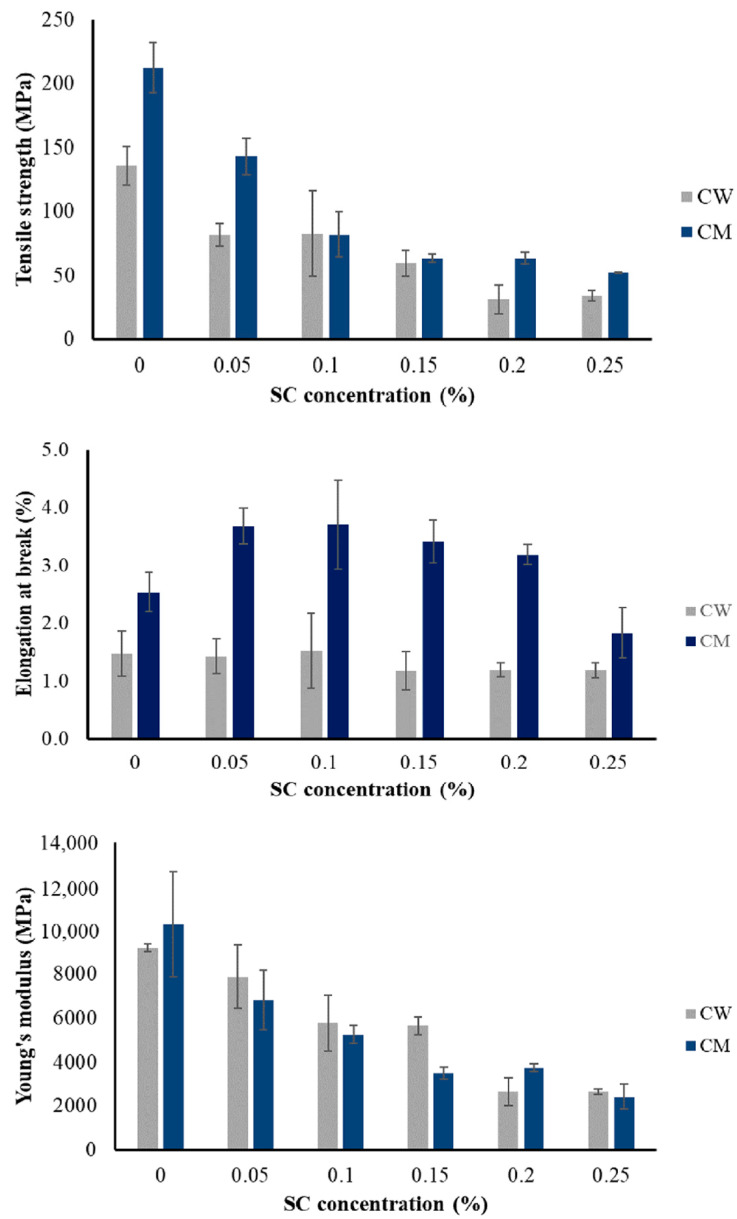
Mechanical properties of dried BC films prepared from mature coconut water (CW) and coconut skim milk (CM) culture medium supplemented with silk at 0.00–0.25%.

The mechanical properties of the films were measured in terms of tensile strength, Young’s modulus, and elongation at break, as shown in Figure 3. The thickness of BCW and BCM was around 0.025 mm, and the thickness of S/BCM and S/BCW was 0.040–0.050 mm. The tensile strength, Young’s modulus, and the elongation at the break of BCM were 212.4 MPa, 10,268.3 MPa, and 2.54%, respectively, whereas the tensile strength, Young’s modulus, and the elongation at the break of BCW were 135.9 MPa, 9184.0 MP, and 1.47%, respectively. The tensile strength of BCM was significantly higher than that of BCW, approximately 56%, whereas the elongation at break of BCM was about 73% higher than that of BCW. However, an insignificant difference in Young’s modulus of BCM was observed when compared with that of BCW. BCM could resist higher force tension, which might be due to its thicker layers of sheet and denser structure. The addition of silk could affect the formation and arrangement of fiber layers, resulting in significant reductions of tensile strength and Young’s modulus of S/BCW and S/BCM owing to the high brittleness of silk fibroin in dry-state [34]. The elongation at break values of S/BCW was in the same range as that of BCW, but by adding silk at 0.05–0.2%, a significant increase in elongation at the break of S/BCM was noticed. Previously, it was reported that fatty acid esters could act as natural plasticizers to reduce the brittleness of PHB-V films [35]. In this work, fat in coconut skim milk could incorporate well into hydrogel, resulting in a more elastic structure and relatively higher elongation at break and tensile strength of S/BCM compared with S/BCW.

### 2.3. Fourier Transform Infrared (FT-IR) Spectra

**Figure 4:**
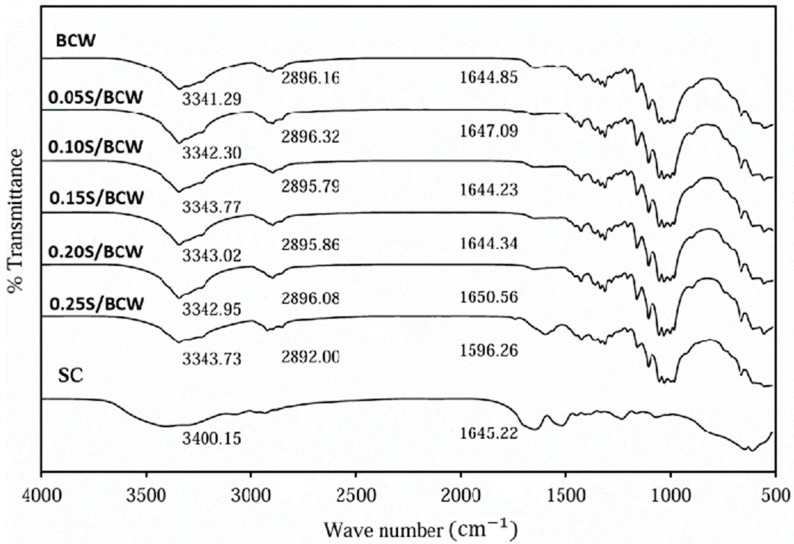
FTIR spectra of SC, BCW film, and S/BCW films.

**Figure 5:**
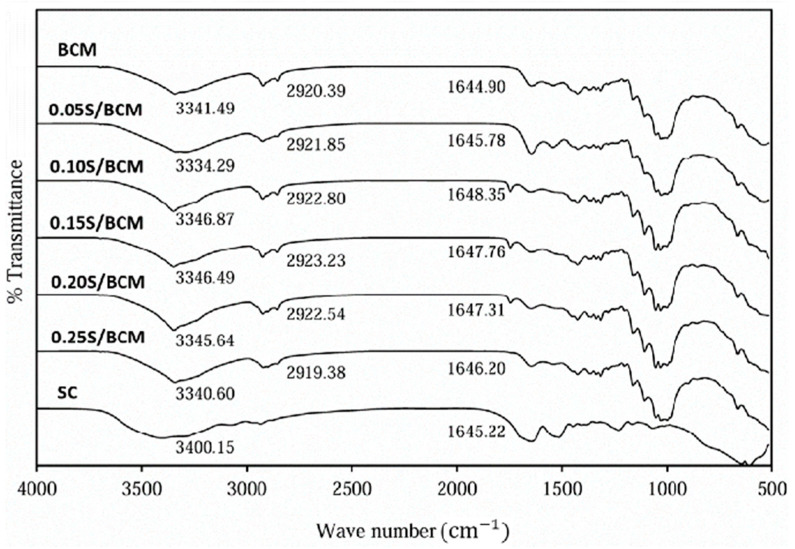
FTIR spectra of SC, BCM film, and S/BCM films.

FTIR analysis was performed to investigate the chemical structure and spectra shift of the BCW, BCM, and the composite films of BC and silk, as shown in Figure 4 and Figure 5. Characteristic bands of BC were observed in BCW and BCM, including O-H stretching at 3341 cm^−1^ and C=O vibration at 1644 cm^−1^ [36]. The peak at around 2896 cm^−1^ matched the stretching vibration of C-H and the asymmetric elongation of CH_2_. FTIR spectra of SC showed characteristic peaks of amide I at 1645 cm^−1^, amide II at 1515 cm^−1^ [37], and amide A and amide B around 3000–3500 cm^−1^ [38]. The composite films of S/BCW and S/BCM significantly maintained the characteristic peaks of BC with shifting of the O-H peaks from 3341 cm^−1^ to 3342–3343 cm^−1^, and the C=O peak significantly shifted from 1644 cm^−1^ to 1650 cm^−1^ in 0.2S/BCW. The characteristic peaks of silk in the composite films were unclear, which might be due to the low content of silk in the composite films and weak interaction between their functional groups without a new chemical bonding.

### 2.4. X-Ray Diffraction (XRD)

**Figure 6:**
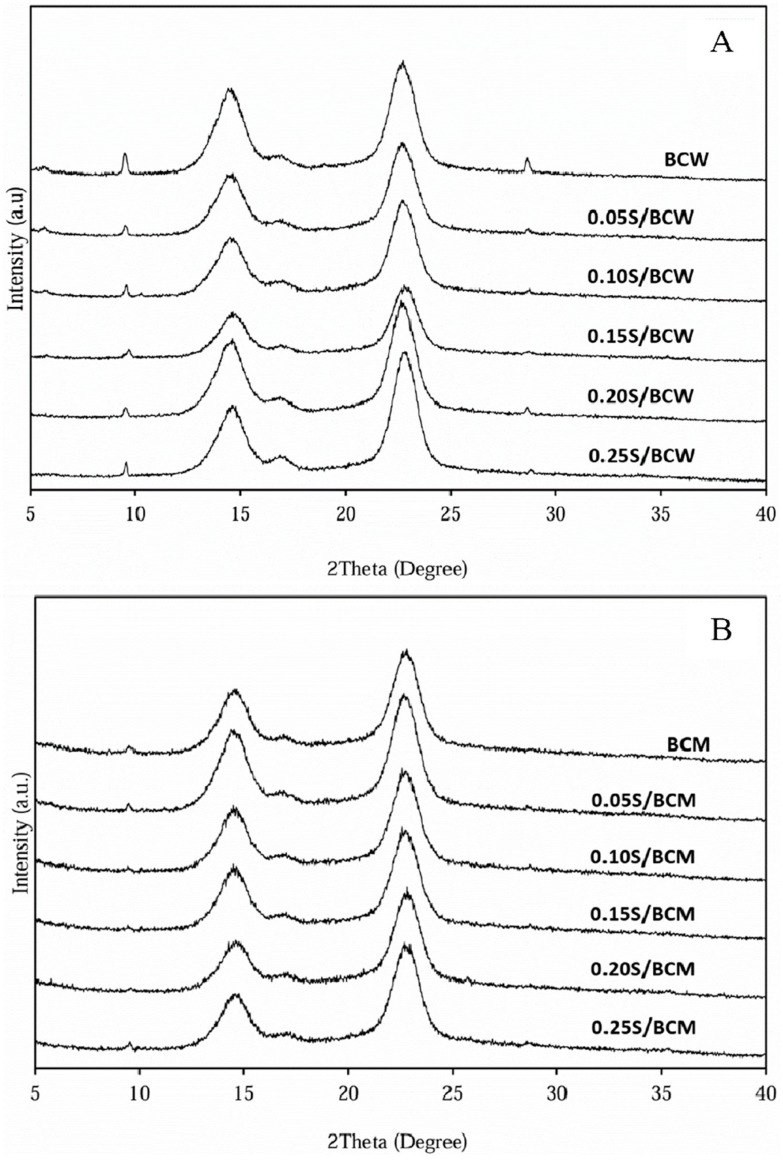
XRD patterns: BCW and S/BCW films (**A**); BCM and S/BCM films (**B**).

The XRD patterns of BC and BC-silk composites are shown in Figure 6. BC takes the cellulose Iα crystal form, with a different unit cell than the Iβ of higher plants. Thus, the corresponding planes are (100), (010), and (110) [39]. The XRD peaks of BCW and BCM consisted of three main peaks at 2 theta, 14.7°, 16.9°, and 22.8°, representing a typical cellulose profile from the BC cultured in static circumstances [40]. However, the degree of crystallinity of BCW was relatively higher than that of BCM (Table 1). According to BCM cross-sectional morphology (Figure 2E, bottom), it was suggested that components in coconut skim milk might obstruct the fiber formation and arrangement, leading to a lower crystalline area in BCM compared with those of BCW. Silk had a very low degree of crystallinity (7.1%); the integration of silk in BCW and BCM affected their degree of crystallinity to some extent. Nonetheless, the crystallinity of BCM and BCW was still in a range of 60–75%, because only a small amount of silk was added into the biosynthesis medium.

### 2.5. Water Absorption Capacity (WAC)

WAC results are summarized in Table 1. The film of BCW shows the highest WAC (630%) due to its high hydrophilicity and its excellent porous structure, while the WAC of BCM was 530%. The WAC of S/BCW and S/BCM films were in the range of 400–500%. According to the components in coconut skim milk (protein, fat, etc.), BCM had a higher hydrophobicity than BCW. Therefore, the WAC of BCM was relatively lower. For S/BCW and S/BCM, because of silk-filled pores and the covered surface of BC, the porosity of films was relatively reduced, leading to a decrease in WAC. As previously noticed in BC/silk fibroin sponge, the reduction in BC pores by silk fibroin addition also caused a decline in WAC [1]. Generally, the fluid absorption capacity of commercial absorbent dressings was found in a range of 50–220% [42,43]. According to the values of WAC of BCW, S/BCW, BCM, and S/BCM, the BC-silk composite films developed in this work could have potential application as absorbent dressing.

### 2.6. Cytotoxicity

**Figure 7:**
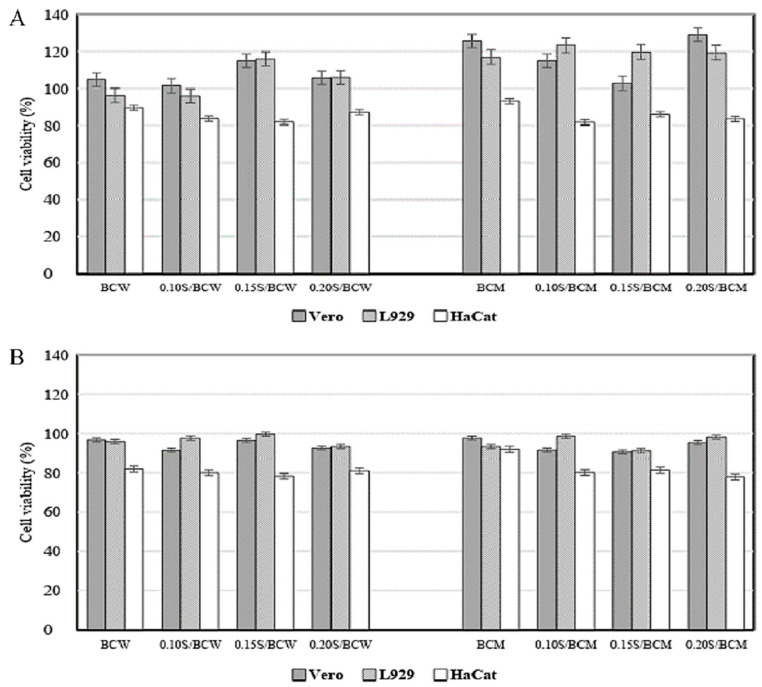
Toxicity test against Vero, L929, and HaCat cell lines on BCW, S/BCW, BCM, and S/BCM films for (**A**) 24 h and (**B**) 48 h.

The cytotoxicity of the composite films was characterized using an indirect method against Vero, L929, and HaCat cells for 24 h (Figure 7A) and 48 h (Figure 7B). The films were immersed in the DMEM medium to extract soluble components from the films. The concentration of living cells cultured with the film extract was compared with that cultured with pure DMEM medium. After 24 h, it was clear that the extracts from BCM and S/BCM promoted Vero and L929 cell growth with cell viabilities of approximately 103–128%, which were slightly higher than those from BCW and S/BCW (96–116%), revealing their nontoxicities and good biocompatibilities. The cell viability of Vero and L929 was still high after 48 h of cultivation with the film extract. The components extracted from the BCM and S/BCM films could be protein, fat, etc. Proteins such as albumin and lysine have been commonly used as a supplement in culture media to enhance the growth of mammalian cells. Silk sericin and fibroin also promoted the proliferation of several mammalian cell lines [44,45]. The cell viability of HaCat cells cultured with the extracts for 24 and 48 h was 80–97%, which was relatively lower as compared to that of Vero and L929 cells. If cell viability was more than 80% relative to that of the nontreated cells, the tested material was considered noncytotoxic. Therefore, all the films in this work were noncytotoxic against Vero, L929, and HaCat cells.

### 2.7. Biocompatibility

**Figure 8:**
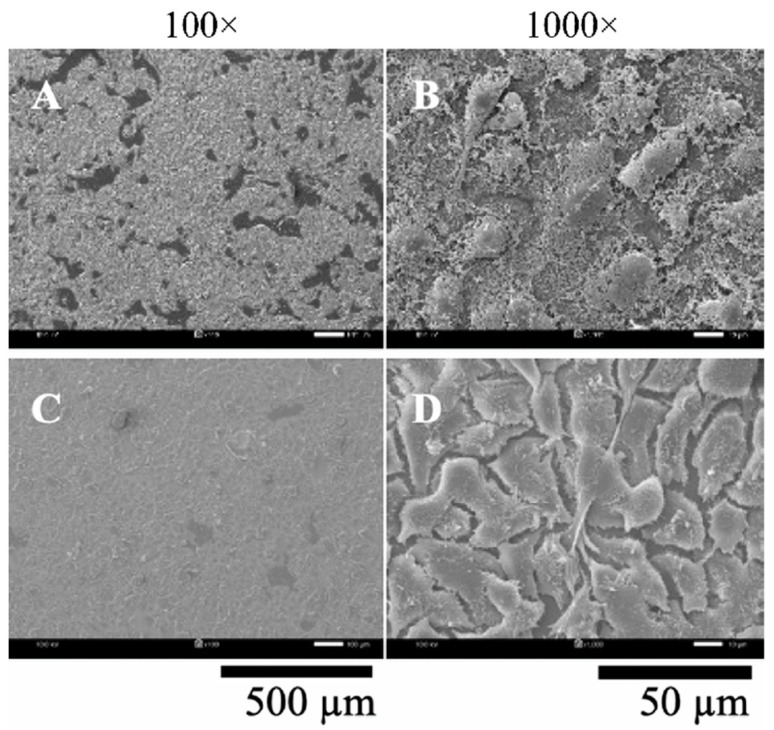
SEM images of Vero (**up**) and HaCat (**down**) cell adhesion on the cover glass (control) for 48 h at magnification of (**A**,**C**) 100×and (**B**,**D**) 1000×.

BCW, BCM, S/BCW, and S/BCM films were tested for their biocompatibility with Vero and HaCat cells using a cover glass slide as a control. SEM images of the cells cultured on the glass slide for 48 h are shown in Figure 8. Both cells attached and proliferated almost entirely on the glass slide surface. Vero cells proliferated and connected with each other with lots of pseudopodia. HaCat cells grew in multi-layers with low connection between the cells.

**Figure 9:**
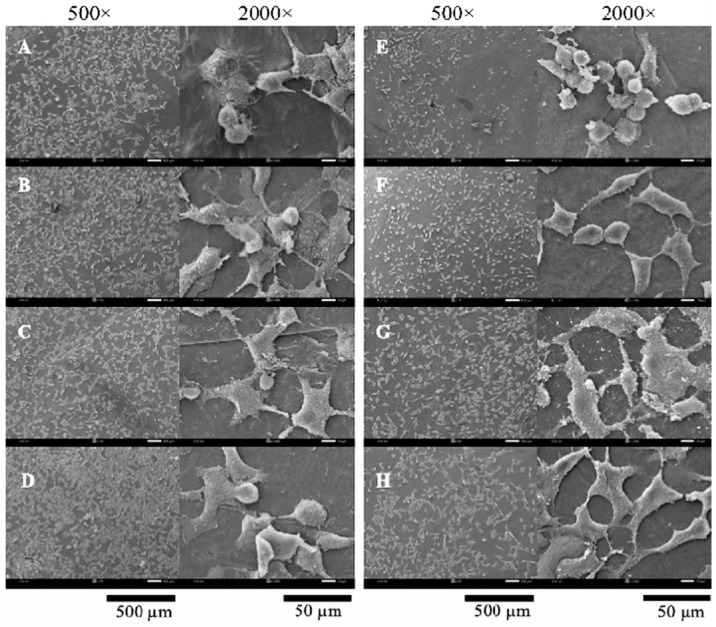
Vero cell adhesion on (**A**) BCW, (**B**) 0.10S/BCW, (**C**) 0.15S/BCW, (**D**) 0.20S/BCW, (**E**) BCM, (**F**) 0.10S/BCM, (**G**) 0.15S/BCM, and (**H**) 0.20S/BCM after cultivation for 48 h.

SEM images of Vero cells grown on the BC composite films are gathered in Figure 9. In comparison, between BCW (Figure 9A) and BCM (Figure 9E), relatively less cell attachment was noticed on the BCM surface without silk supplementation. Low wettability of the material could decrease the cell attachment [46]. According to WAC results, BCW (629.8%) could absorb significantly more water than BCM (534.6%). The rounded morphology of cells was displayed on BCM, indicating a better cell-to-cell interaction than that of the cell-to-matrix [47,48]. The results also demonstrated that with supplementation of silk, cell density gradually increased with the enhancement of silk concentration. Better cell proliferation with many pseudopodia formations was observed on all S/BCW than on BCW. The presence of silk in BCM also significantly promoted cell attachment, proliferation, and cell-to-cell interaction. Rounded cells on BCM gradually stretched their morphology with an increase in silk concentration, and cells started to connect to each other at a silk concentration of 0.15%. Amino acids (glycine, alanine, and serine) contained in silk fibroin were suggested as an important set suitable for application as a controlled-release biomaterial and scaffold for tissue engineering. Previously, by soaking BC in silk fibroin solution, it was shown that the cell attachment on BC/silk fibroin sponge surface was better than on the pure BC one, suggesting that the presence of fibroin improved cell attachment of L929 cells [1]. Sericin, another form of protein in silk, was reported to promote the growth of L929 fibroblasts [34,38].

**Figure 10:**
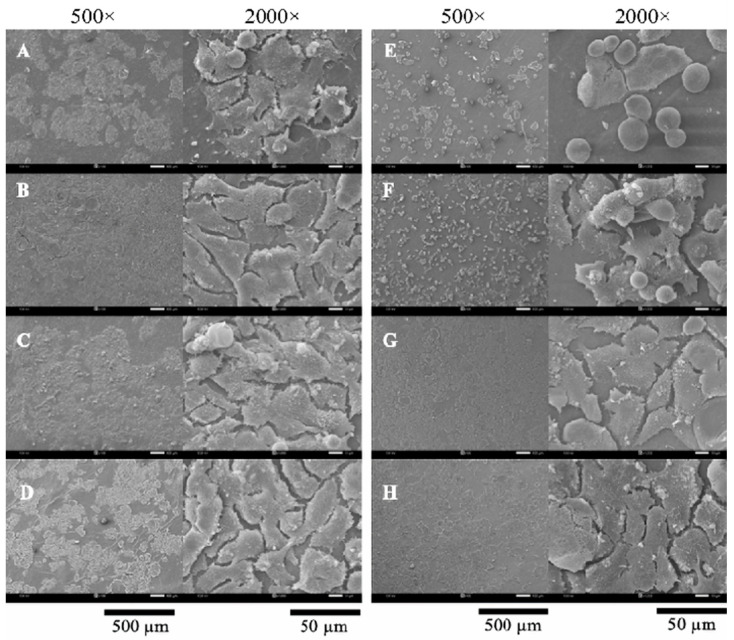
HaCat cell adhesion on (**A**) BCW, (**B**) 0.10S/BCW, (**C**) 0.15S/BCW, (**D**) 0.20S/BCW, (**E**) BCM, (**F**) 0.10S/BCM, (**G**) 0.15S/BCM, and (**H**) 0.20S/BCM after cultivation for 48 h.

For HaCat cells cultured on the films (Figure 10), similar phenomena were observed on BCW and BCM. Without the supplementation of silk, the cells cultured on BCW showed better attachment and proliferation with a higher cell density than those on BCM. Round-shaped cells were noticed more on BCM. Cell density substantially increased on 0.1S/BCW, and the cell almost completely spread over the surface. The incorporation of silk into BCM also obviously promoted the growth, adhesion, and proliferation of HaCaT cells. Pseudopodia started to form on 0.1S/BCM surface. Cells became fully spread on 0.15S/BCM and 0.2S/BCM. Cell density on S/BCM was increased with the increase in silk concentration. Therefore, the supplementation with silk in the form of thin-shell silk cocoon powder in the culture medium in the biosynthesis of BC films could promote cell adhesion and proliferation on the composite films. Previously. it was reported that the integration of silk fibroin into polycaprolactone could promote the cell growth and proliferation of human adipose-derived mesenchymal stem cells (hADMSC) [49]. When the silk cocoon membrane was exposed to water, the water molecules diffused into its porous matrix and interacted with ion-forming elements like Na, K, and Cl, as well as the protein matrix, resulting in the generation of charged ionic species [50]. The attachment of cells to the material was influenced by the physicochemical properties of interactions such as ionic and van der Walls forces [51]. The cell membrane had negative charges, simply binding with positive charges on the material surface [52,53,54].

## 3. Conclusions

The S/BC composite films were fabricated through simple techniques using coconut water/coconut skim milk as a cost-effective culture medium. The powder of thin-shell silk cocoons, a byproduct from the silk textile industry, was used as the source of silk. Compared with BCW film, the structure of BCM film displayed a less orderly and denser structure. It was suggested that the components contained in coconut skim milk (protein, fat, etc.) could partially disturb the nanocellulose fiber synthesis of the bacteria *A. xylinum*. From the incorporation of silk into BCW and BCM, the surface became smoother, and the fiber layers became denser with increasing silk concentration. Because of the high brittleness of silk, the mechanical strength of S/BCW and S/BCM films was relatively lower than that of the BC films without adding silk. However, it was demonstrated that fat in coconut skim milk could incorporate well with silk and act as a natural plasticizer to enhance the elasticity of S/BCM films. The integration of silk into the BC matrix reduced the WAC of the composite films to some extent; nonetheless, the values of WAC of S/BCW and S/BCM were in the high range of absorbent dressings. In addition, the composite films of S/BCW and S/BCM were nontoxic against Vero, L929, and HaCat cells. Better cell attachment and proliferation with good cell-to-cell interaction were noticed on the surfaces of films supplemented with silk. For Vero and HaCat cells, the silk concentrations of 0.15–0.20% (*w*/*v*) for the BC biosynthesis significantly promoted cell adhesion and cell-to-cell interaction on the BCW and BCM films. From the results, it can be concluded that the composite films of BCM and BCW incorporated with silk have potential properties that are promising for application as wound dressings and scaffolds for tissue engineering.

## 4. Materials and Methods

### 4.1. Materials

Thin-shell silk cocoon was obtained from the Queen Sirikit Department of Sericulture, Saraburi, Thailand. The thin-shell silk cocoon was fragmented into 140 mesh using a ball mill and was kept in a desiccator until used. The average size of silk cocoon powder particles was about 0.1 mm. Nitrogen, carbon, and hydrogen contents in silk powder determined by Elemental analyzer (CHNS) were 16.13%, 43.64%, and 6.44%, respectively [13]. The stock culture of *Acetobacter xylinum* (AGR60) was kindly provided by Pramote Thamarat (Institute of Research and Development of Food Product, Kasetsart University, Bangkok, Thailand). The tests for cytotoxicity and biocompatibility were performed by the service from Scientific Instruments Center KMITL (Sci-Ins KMITL), Faculty of Science, King Mongkut’s Institute of Technology (Ladkrabang District, Bangkok, Thailand) using African green monkey kidney fibroblast (Vero): CLS605372, mouse subcutaneous connective tissue (L929): CLS400260, and human keratinocyte immortal cells (HaCat).

### 4.2. Fabrication of BC and S/BC Films

**Figure 11:**
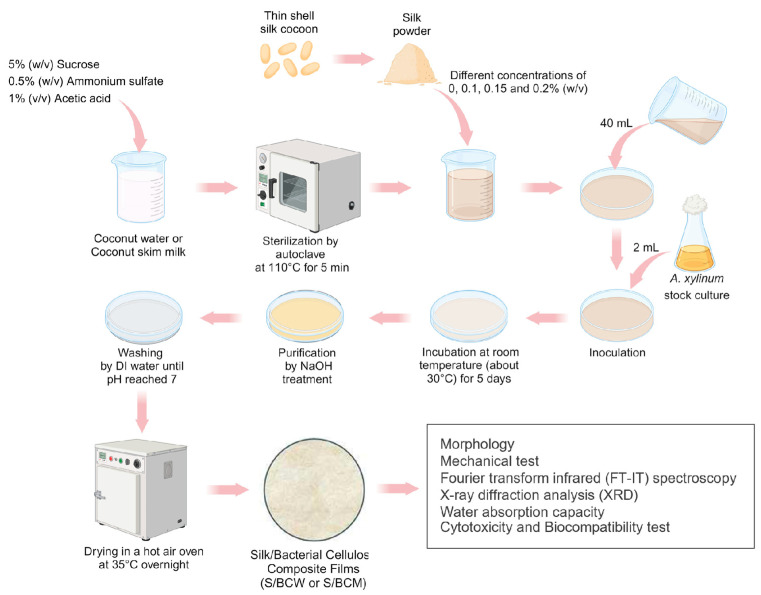
Schematic diagram of the fabrication process of BC and S/BC films.

The culture medium for film preparation via biosynthesis technique was mature coconut water or coconut skim milk supplemented with 5% *w*/*v* sucrose, 0.5% *w*/*v* ammonium sulfate, and 1% *v*/*v* acetic acid [55]. The media were sterilized at 110 °C for 5 min. Stock culture of *Acetobacter xylinum* (AGR60) of 2 mL was added into 40 mL of the sterilized medium in a 14 cm diameter Petri dish and then was incubated at room temperature (about 30 °C) for 5 days. For S/BC film, thin-shell silk cocoon powder was added into the sterilized medium in different concentrations of 0, 0.1, 0.15, and 0.2% *w*/*v*, which are marked as (for films prepared from coconut water) BCW, 0.1S/BCW, 0.15S/BCW, and 0.2S/BCW, respectively, and (for films prepared from coconut skim milk) BCM, 0.1S/BCM, 0.15S/BCM, and 0.2S/BCM, respectively. After the pellicles were formed, they were purified by rinsing with DI water and treating with 1% *w*/*v* sodium hydroxide solution for 24 h. Then, they were rinsed with DI water until pH reached 7. Finally, the purified pellicles were dried in a hot air oven at 35 °C overnight to obtain the composite films. The summarized procedure for the fabrication process of BC and S/BC films is shown in Figure 11.

### 4.3. Morphology

The morphological structure of BC and S/BC films was analyzed by Field Emission Scanning Electron Microscopy, FESEM (JEOL JSM-7610F, Tokyo, Japan). The accelerating voltage was controlled at 5 kV.

### 4.4. Mechanical Test

The mechanical tests of BC and S/BC films were performed by Instron Universal testing machine (UTM) following ASTM D882 guidelines. The samples were cut into strip-shaped specimens of 10 mm in width and 10 cm in length. Five specimens were used for each condition.

### 4.5. Fourier Transform Infrared (FT-IR) Spectroscopy

The chemical functional group and interaction between BC and thin-shell silk cocoon were analyzed by Nicolet FTIR Spectrometer (SX-170, Thermo fisher scientific, Waltham, MA, USA). The FTIR analysis was performed by the technicians at the Scientific and Technological Research Equipment Centre, Chulalongkorn University (STREC CHULA, Bangkok, Thailand, https://strec.chula.ac.th, accessed on 1 November 2024). The FT-IR spectra were collected in the range of 4000–500 cm^−1^ at a resolution of 1 cm^−1^.

### 4.6. X-Ray Diffraction (XRD) Analysis

X-ray diffraction characterization of crystallinity was conducted by the technician at the Scientific and Technological Research Equipment Center, Chulalongkorn University (STREC CHULA, Bangkok, Thailand, https://strec.chula.ac.th, accessed on 1 November 2024). X-ray patterns were obtained using an X-ray diffractometer (XRD, Bruker AXS Model D8 Discover, Karlsruhe, Germany) equipped with VA° NTEC-1 super speed detector under the voltage of 40 kV, at a current of 40 mA and angle (2θ) of 5–40°. The relative intensity was recorded in a step of 0.02°. The degree of crystallinity of composite films was calculated using the ratio of the crystalline peak areas and the total XRD profile area after subtracting the background intensity acquired by TOPAS Rietveld analysis software. Rietveld analysis of the XRD data using the fundamental parameters approach (FPA) for peak fitting and the Chebychev polynomial for background subtraction [56]. The Rietveld method optimizes variables to fit a diffraction pattern, and it uses all of the diffraction peaks [39]. Profile fitting and crystallinity (%) calculations were performed with TOPAS (version 3.0) software.

(1) $$\mathrm{Degree}\;\mathrm{of}\;\mathrm{crystallinity}\;(\%)=\frac{Crystalline\;area\;\times \;100}{Total\;Area}$$

### 4.7. Water Absorption Capacity

The dried films were weighted (W_d_) before they were immersed in DI water at room temperature until equilibrium. Then, the swollen films were removed from water and blotted out with Kim wipes. The weight of the swollen film (W_h_) was measured. WAC was calculated using the following formula:

(2) $$WAC=\frac{\left(W_{h}-W_{d}\right)}{W_{d}}\times 100$$

### 4.8. Cytotoxicity Test

The cytotoxicity of composite films was determined using mouse fibroblast (L929 cells) and African green monkey kidney fibroblast (Vero cells). To obtain the film extract, the films were soaked in Dulbecco’s modified eagle medium (DMEM) and incubated at 37 °C for 24 h. The L929 and Vero cells of 1 × 10^5^ cells/mL were cultured in a 96-well plate with 100 μL of DMEM in a CO_2_ incubator at 37 °C, 5% CO_2_ for 24 h. After that, the culture medium in each well was replaced by 100 μL of 1000 μg/mL of the film extract and then was further incubated at 37 °C for 24 and 48 h. The 3-(4,5-dimethylthiazolyl-2)-2,5-diphenyltetrazolium bromide (MTT) assay was used to quantify the number of living cells. The extract was firstly removed, and then 10 μL of 5 mg/mL MTT solution was added into each well and then incubated at 37 °C, 5% CO_2_ for 4 h. Afterward, MTT solution was removed, and 150 μL of dimethyl sulfoxide (DMSO):10% SDS (in a ratio of 9:1) was added into each well. Then, the sample was shaken for 5 min before the optical density (OD) was measured by a microplate reader (EZ Read 2000, biochrom, Cambridge, UK) at a wavelength of 570 nm.

### 4.9. Biocompatibility Test

The BC film and S/BC film were cut to a circle-shape of 15 mm in diameter and were sterilized at 121 °C for 25 min. The sterilized films were placed on a 24-well tissue culture plate, and a metallic ring was placed on top. After that, 0.5 mL of DMEM containing 10% Fetal bovine serum (FBS), 100 U/mL Penicillin, and 100 μg/mL Streptomycin was added into each well and then incubated at 37 °C, 5% CO_2_ for 1 h. The DMEM was removed, and 2 × 10^5^ cells of Vero or HaCat cells were seeded on the film. The incubation was performed for 48 h to examine the cell attachment and proliferation using FESEM.

### 4.10. Statistical Analysis

Statistical analysis was presented as mean ± the standard deviation (SD) and obtained from at least three independent tests. All data were analyzed using Minitab 17 software. Error bars represent SD. The differences were considered to be statistically significant at the value of *p* < 0.05.

## Figures and Tables

**Table 1:** Crystalline and amorphous areas, degree of crystallinity, and water absorption capacity [41] of dried films of BCW, S/BCW, BCM, S/BCM, and silk (S).

Sample	Crystalline Area	Amorphous Area	Degree of Crystallinity * (%)	WAC ** (%)
BCW	8208	2501	76.65	629.79 ± 0.12
0.10S/BCW	6295	2159	74.46	493.38 ± 0.09
0.15S/BCW	4597	2346	66.21	483.19 ± 0.05
0.20S/BCW	6518	2456	72.63	397.30 ± 0.02
BCM	4821	2515	65.72	534.65 ± 0.03
0.10S/BCM	5707	2632	68.44	480.38 ± 0.08
0.15S/BCM	4586	2423	65.43	451.695 ± 0.05
0.20S/BCM	4207	2847	59.64	421.85 ± 0.13
S	577.2	7457	7.18	-

* Degree of crystallinity = Crystalline area/Total area. ** Data were the averages for 5 specimens.

## Data Availability

The original contributions presented in the study are included in the article. Further inquiries can be directed to the corresponding author.
